# Quantification of Biologically Active DNA Alkylation in Temozolomide-Exposed Glioblastoma Cell Lines by Ultra-Performance Liquid Chromatography–Tandem Mass Spectrometry: Method Development and Recommendations for Validation

**DOI:** 10.1021/acsomega.3c01818

**Published:** 2023-06-23

**Authors:** Margaux Fresnais, Ina Jung, Uli B. Klein, Dirk Theile, Siwen Liang, Walter E. Haefeli, Jürgen Burhenne, Rémi Longuespée

**Affiliations:** Department of Clinical Pharmacology and Pharmacoepidemiology, Heidelberg University Hospital, Im Neuenheimer Feld 410, 69120 Heidelberg, Germany

## Abstract

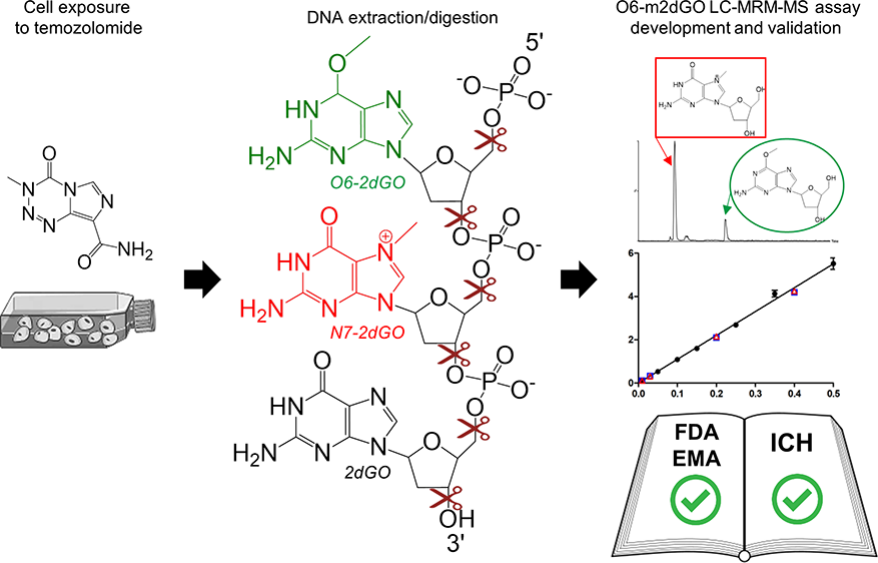

Quantitative monitoring of biologically active methylations of guanines in samples exposed to temozolomide (TMZ) would be useful in glioblastoma research for preclinical TMZ experiments, for clinical pharmacology questions regarding appropriate exposure, and ultimately for precision oncology. The known biologically active alkylation of DNA induced by TMZ takes place on O6 position of guanines. However, when developing mass spectrometric (MS) assays, the possible signal overlap of O6-methyl-2′-deoxyguanosine (O6-m2dGO) with other methylated 2′-deoxyguanosine species in DNA and methylated guanosines in RNA must be considered. Liquid chromatography–tandem MS (LC–MS/MS) offers the analytical requirements for such assays in terms of specificity and sensitivity, especially when multiple reaction monitoring (MRM) is available. In preclinical research, cancer cell lines are still the gold standard model for in vitro drug screening. Here, we present the development of ultra-performance LC-MRM-MS assays for the quantification of O6-m2dGO in a TMZ-treated glioblastoma cell line. Furthermore, we propose adapted parameters for method validation relevant to the quantification of drug-induced DNA modifications.

## Introduction

Mass spectrometric (MS) quantification of temozolomide (TMZ)-induced DNA modifications would represent an ideal pharmacodynamic approach to monitor TMZ action in precision oncology and drug development for glioblastoma (GBM).^1^ Among DNA modifications triggered by TMZ, the methylation of 2′-deoxyguanosine (2dGO) at the O6 position is the known one mainly responsible for TMZ action.^1^ O6-methyl-2′-deoxyguanosine (O6-m2dGO) can be repaired by the methylguanine methyl transferase (MGMT), which is unfunctional in a majority of GBM patients, especially in the presence of mutations of isocitrate dehydrogenase (IDH).^1^ IDH mutations lead to epigenetic changes that can inactivate the promoter of MGMT.^1^ The expression of MGMT is therefore silenced, thus preventing the reparation of O6-m2dGO.^1^ With a functional mismatch repair machinery, O6-m2dGO will be replaced by thymine. As an effect, DNA double strand breaks (causing autophagy) and/or futile cycling (promoting cell death) will take place. Although the quantification of O6-m2dGO would allow to evaluate the biologically active chemical modifications of DNA induced by TMZ, it is challenged by the existence of a large majority of other alkylated species such as N7-methyl-2′-deoxyguanosine (N7-m2dGO) in DNA and methylated guanosines in RNA. Indeed, O6-m2dGO represent only 5–10% of the nucleoside alkylation in DNA, while TMZ also leads to the alkylation of guanines at N7-position.^1^ N7-m2dGO is repaired by the base excision repair machinery, which is rarely inactive in GBM.^1^ N7-m2dGO is therefore biologically inactive but represents 60–80% of nucleoside alkylation induced by TMZ. N7-m2dGO represents a major analytical issue for the quantification of O6-m2dGO since both species present the same mass, as well as their fragments, N7-methylguanine (N7-mG) and O6-methylguanine (O6-mG), respectively.^1^ In addition, alkylation induced by TMZ is not specific to DNA but also affects RNA. When analyzing methylated guanines, the presence of alkylated RNA prevents the specific quantification of methylated guanines from DNA without a sample preparation including RNA removal.^1,2^ Since reducing sample preparation steps would be critical for future sensitive analysis of low-volume samples (e.g., low cell counts of limited tissue pieces), analysis of O6-m2dGO would thus be the most suited strategy to potentially skip the RNA removal step. We have recently demonstrated that using desorption/ionization MS methods would only be possible with instruments equipped with ion mobility and specific high-resolution (HR) settings that would efficiently separate O6 and N7 species of methylated guanines and 2dGO.^3^ In this context, the analysis of nucleosides by ultra-performance liquid chromatography (UPLC)-tandem mass spectrometry (MS/MS) seems to be the most appropriate method to separate O6- and N7-methylated species based on their difference of hydrophobicity. Analyses in multiple reaction monitoring (MRM) mode allow for the selection and fragmentation of compounds with high specificity.^4−6^ DNA digestion for the selective recovery of nucleosides provides more flexibility for future development of rapid methods while avoiding an RNA removal step.^1^ In preclinical neuro-oncological in vitro studies, methods for drug quantification and evaluation of drug effects in cell lines are still required. Since TMZ is still the standard of care in GBM, it is also considered in combination with newly developed drugs for in vitro and in vivo assays as well as in clinical trials.^7−9^ Moreover, intensive research is still ongoing to investigate the actual effect of TMZ in GBM.^9^ Here, we present method developments for the specific quantification of O6-m2dGO in the GBM cell line LN229 exposed to TMZ and the evaluation of the O6-m2dGO/2dGO ratio as a potential marker for TMZ action.^10^ Since the compounds to be quantified are incorporated into the complex structure of DNA, the generation of calibration curves and preparation of quality controls (QCs) using classical procedures for drug quantification is not directly possible. Consequently, bioanalytical method validation (BMV) strategies should be adapted as specified in regulatory guidelines^11^ to ensure method reliability and robustness for future applications. In the present article, we describe such adapted strategies for the development of an (i) informative, (ii) cost-efficient, and (iii) time-efficient bioanalytical assay for the assessment of TMZ action in cell lines through the quantification of O6-m2dGO.

This study may also serve as a first step toward the quantification of methylated guanosines in clinical samples. With appropriate adjustments, particularly with respect to sample scale (i.e., lower cell numbers), such quantifications are expected to be possible in human GBM tissues.

## Materials and Methods

### Chemicals

MS-grade water, organic solvents, and formic acid (FA) were purchased from Biosolve Chimie SARL (Dieuze, France). 2dGO, O6-m2dGO, 2′-deoxy-N-ethyl-guanosine (2dNetGO), d3-O6-methyl-2′-deoxyguanosine (d3-O6-m2dGO), N7-mG, O6-mG, and N2-methyl-2′-deoxyguanosine (N2-m2dGO) were purchased from Toronto Research Chemicals (North York, Canada) (Supporting Information, Figure S1). Nucleoside Digestion Mix NEB #M0649 was purchased from New England Biolabs (New England Biolabs, Ipswich, MA, USA). Cell culture medium, Dulbecco’s Modified Eagle’s Medium (DMEM), and fetal calf serum (FCS) were purchased from PAN-Biotech (Aidenbach, Germany). Glutamine and penicillin/streptomycin were purchased from Sigma-Aldrich (Taufkirchen, Germany). Crystal violet was purchased from Applichem (Darmstadt, Germany). The bromodeoxyuridine (BrdU) assay kit was purchased from Merck (Darmstadt, Germany). The DNA extraction kit was purchased from Qiagen (Hilden, Germany).

### Standard Solution Preparation

Stock and sub-stock solutions of the different compounds were prepared as described in the Supporting Information, section “Supplementary Materials and Methods”.

**Figure 1:**
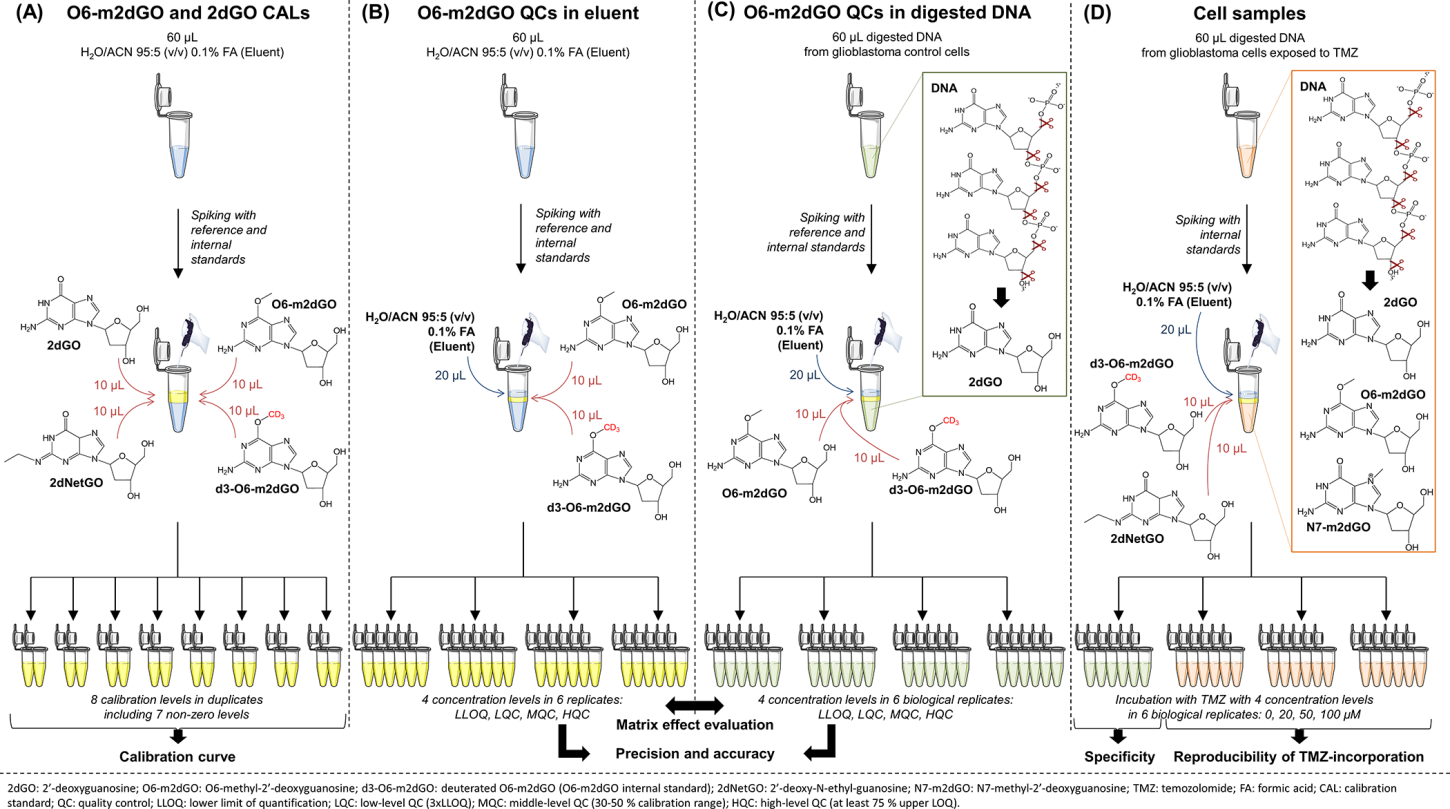
Calibration standard (CAL) (A), quality control (QC) (B, C), and cell sample preparation (D) for method prevalidation and validation batches of the O6-m2dGO quantification assay to evaluate all required parameters, such as linearity range (A), lower limit of quantification (A–D), precision and accuracy (B, C), matrix effect reproducibility (B, C), specificity (D), and reproducibility of TMZ incorporation (D).

For the preparation of calibration standards (CAL) and QC samples in LC eluent (H_2_O/ACN 95:5 (v/v) 0.1% FA), separate dilution series was prepared from the 1 μg/mL sub-stock solution of O6-m2dGO and from the 1.56 mg/mL stock solution of 2dGO as described in Table 1 (reference standard solutions). For each calibration level, 10 μL of each reference standard solution (2dGO and O6-m2dGO) at the corresponding concentration was added to 10 μL of each internal standard (IS) solution (0.600 ng/mL d3-O6-m2dGO and 600 ng/mL 2dNetGO) and the volume was filled up to 100 μL with LC eluent (H_2_O/ACN 95:5 (v/v) 0.1% FA) (Figure 1A,B). Virtual concentrations in digested DNA are summarized in Table 1, and IS concentrations were fixed at 0.100 and 100 ng/mL for d3-O6-m2dGO and 2dNetGO, respectively.

**Table 1:** In-Solution and Virtual In-Sample Concentrations of Calibration Standard and Quality Control Series of O6-m2dGO and 2dGO for Initial Tests and Prevalidation and for Method Validation^a^

	initial concentration ranges (ng/mL)				validation concentration ranges (ng/mL)			
	O6-m2dGO		2dGO		O6-m2dGO		2dGO	
	solution	sample	solution	sample	solution	sample	solution	sample
CAL H	3.000	0.500	3000	500				
CAL G	2.100	0.350	1500	250	3.000	0.500	3000	500
CAL F	1.500	0.250	600	100	2.100	0.350	1500	250
CAL E	0.900	0.150	300	50	1.500	0.250	600	100
CAL D	0.600	0.100	150	25	0.900	0.150	300	50
CAL C	0.300	0.050	60	10	0.600	0.100	150	25
CAL B	0.060	0.010	30	5	0.300	0.050	60	10
CAL A	0.030	0.005	6	1	0.060	0.010	30	5
HQC	2.400	0.400			2.400	0.400		
MQC	1.200	0.200			1.200	0.200		
LQC	0.090	0.015			0.180	0.030		
LLOQ	0.030	0.005			0.060	0.010		

a O6-m2dGO: O6-methyl-2′-deoxyguanosine; 2dGO: 2′-deoxyguanosine; CAL: calibration standard level; HQC: high-level QC; LLOQ: lower limit of quantification; LQC: low-level QC; MQC: middle-level QC; QC: quality control.

### Biological Assays and Preparation of Cell-Based Standards and Samples

The assay was developed using the LN229 GBM cell line.^10^ The cells were cultured under standard conditions with DMEM, supplemented with 10% FCS, 2 mM glutamine, 100 U/mL penicillin, and 100 μg/mL streptomycin sulfate.

Prior to developing the analytical method, biological effects of TMZ on LN229 cells were evaluated according to our previously published protocol using crystal violet staining,^12^ as described in the Supporting Information, section “Supplementary Materials and Methods”.

Accordingly, for method prevalidation and validation, LN229 cells were exposed for 6 h to 20, 50, or 100 μM TMZ, and each concentration level was prepared in six biological replicates. Control samples of LN229 cells were prepared similarly but without TMZ and were used as control cell samples or to prepare the biological matrix for CALs and QCs for method development and validation steps. After cell trypsinization, DNA was extracted using the Qiagen extraction kit according to the manufacturer’s instructions. DNA was eluted from the column with 100 μL of H_2_O and quantified by absorption spectroscopy (Eppendorf, Hamburg, Germany). To ensure comparable results, a volume containing 5 μg of DNA was processed for each sample. The 5-μg aliquot of extracted DNA was digested using the Nucleoside Digestion Mix by adding 6.5 μL of 10x digestion buffer, 3.25 μL of the enzyme mix, and a volume of H_2_O specific to each sample to reach a final volume of 65 μL. The solution was incubated for 60 min at 37.5 °C to perform the DNA digestion.

From the total final sample volume (i.e., 65 μL), 60 μL was transferred in the LC vials. For CALs and QCs prepared in digested DNA from control cells, 10 μL of O6-m2dGO reference standard solution, 10 μL of d3-O6-m2dGO IS solution, and 20 μL of LC eluent were added to this 60 μL to reach 100 μL (Figure 1C). For TMZ-treated and control cell samples, 10 μL of each IS solution (i.e., d3-O6-m2dGO and 2dNetGO) and 20 μL of LC eluent were added (Figure 1D).

### Liquid Chromatography

Liquid chromatography was performed with a Waters Acquity classic UPLC system (Waters Corp, Milford, MA, USA). Two types of columns were used for initial tests described in the Supporting Information, section “Supplementary Results” (Selection of the Chromatographic Method): a CORTECS UPLC hydrophilic-interaction liquid chromatography 1.6 μm (Agilent, Santa Clara, CA, USA), and an ACQUITY UPLC BEH C18 1.7 μm (Waters). For further assay development, the C18 column was used. From the sample volume of 100 μL, a total of 20 μL was injected in the loop using the full loop mode before transfer onto the analytical C18 column equipped with integrated filter disc. UPLC separation was performed using a system of two eluents: (A) H_2_O/ACN 95:5 (v/v) 0.1% FA and (B) ACN 0.1% FA. The flow rate was set to 0.5 mL/min and column temperature to 40 °C. The LC gradient started with initial conditions at 100% A that were maintained for 0.1 min, and then the eluent composition changed to 50% A over 1.9 min and A was further reduced to 5% over 0.2 min. Eluent composition at 5% A was maintained for 0.3 min, and the system was set back to initial conditions over 0.3 min. Initial conditions were further maintained for 0.2 min before the end of the 3-min LC method.

### Mass Spectrometric Analyses and Treatment of Molecular Data

The analyses were performed using a Waters Xevo G2-XS quadrupole-time of flight mass spectrometer for compound characterization in HR (Supporting Information, section “Supplementary Results,” MS And MS/MS Characterization) and a Waters Xevo TQ-XS (triple quadrupole) mass spectrometer both equipped with Z-spray (electrospray) ionization and step-wave source optimization, and controlled under MassLynx v4.2 (Waters) as fully described previously.^13^ The Waters Xevo G2-XS was used for the compounds characterization in HR, in full-scan, and in quadrupole selection mode without or with increasing collision energy up to 35 V for fragment generation. The Waters Xevo TQ-XS was used for the implementation of the MRM method as detailed in Supporting Information, section “Supplementary Results” and Table S1. The final MRM transitions for O6-m2dGO and 2dGO analyses are given in Table 2 with expected LC retention times of each compound and selected MRM transitions for LC method optimization.

**Table 2:** Final Multiple Reaction Monitoring Transitions for the Specific Detection of O6-m2dGO, 2dGO, and Respective Internal Standards (d3-O6-m2dGO and 2dNetGO)^a^

compound	molecular ion (*m/z*)	product ion (*m/z*)	retention time (min)
2dGO	267.82	151.92	0.49
	267.82	134.92	0.49
O6-m2dGO	282.95	165.93	0.92
	281.95	148.90	0.92
2dNetGO	296.01	134.96	0.95
d3-O6-m2dGO	284.95	151.89	0.92
	284.95	168.93	0.92

a 2dGO: 2′-deoxyguanosine; 2dNetGO: 2′-deoxy-N-ethyl-guanosine; d3-O6-m2dGO: d3-labeled O6-m2dGO; O6-m2dGO: O6-methyl-2dGO.

MassLynx and TargetLynx v.4.2 (Waters) were used for rapid chromatogram and spectra evaluation and for the construction of calibration curves and the computing of quantification data, respectively. As only 60 μL out of 65 μL was taken from each sample for analysis and CALs were prepared as 60 μL-samples, calculated concentrations were corrected by a factor 1.083 to retrieve real concentrations in the samples.

## Results

**Figure 2:**
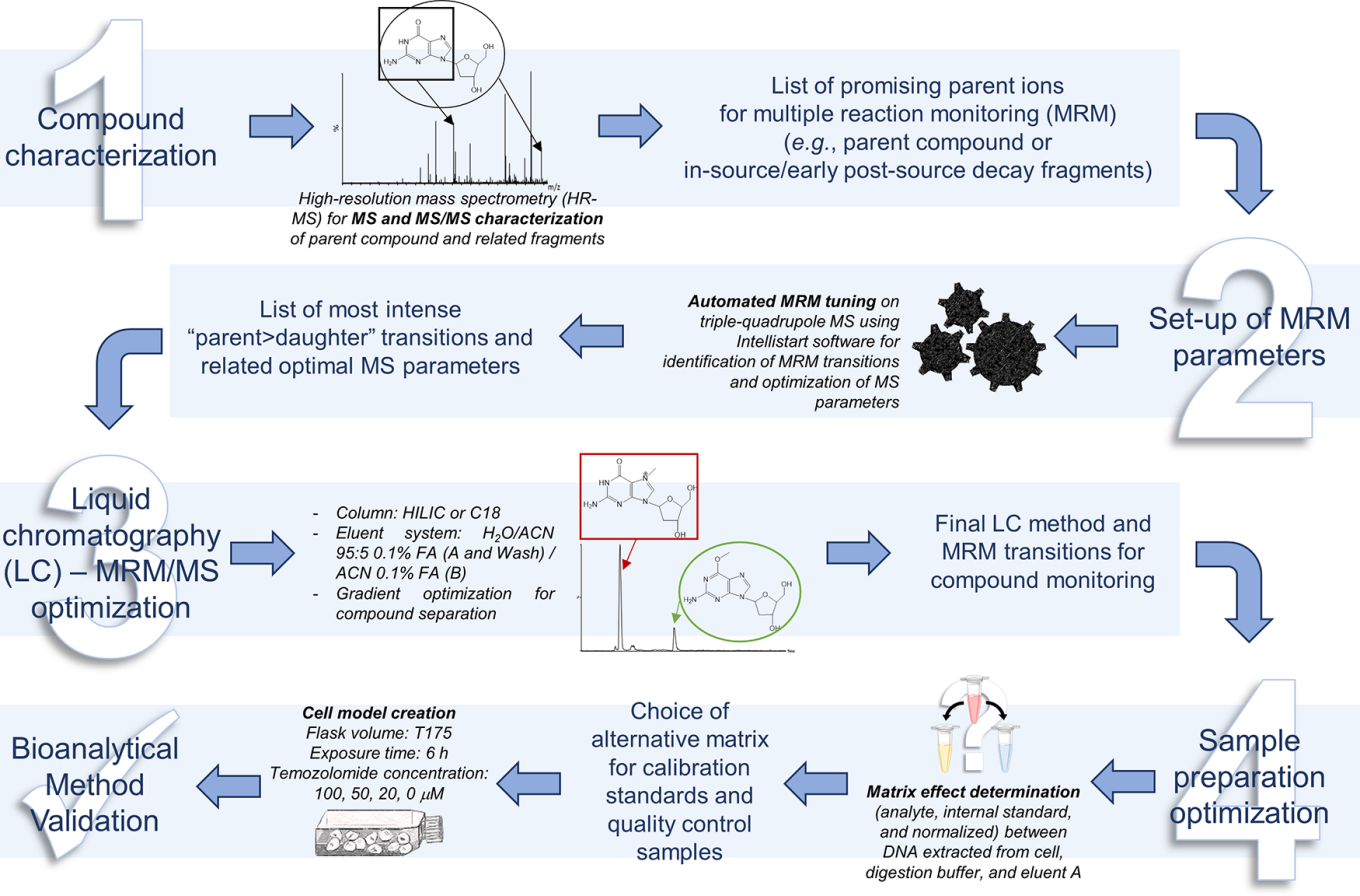
Steps of method development before validation of the bioanalytical assay to monitor TMZ biological action through O6-m2dGO quantification: compound characterization using high-resolution mass spectrometry (HR-MS) (1), multiple reaction monitoring (MRM) mode tuning for MS method set-up (2), optimization of liquid chromatography separation and selection of the final MRM transitions (3), and optimization of the sample preparation (4).

The objectives of this assay development (Figure 2) were (i) to specifically quantify O6-m2dGO in the DNA of a GBM cell line exposed to TMZ and (ii) to measure the ratio of O6-m2dGO/2dGO. The use of MRM mode allows for the highly specific selection of parent and fragment ions for analysis, thus avoiding any contamination with RNA-derived compounds. At the same time, it is critical to separate O6-m2dGO from N7-m2dGO by LC because their masses overlap.

### LC-MRM-MS Method Development and Optimization

**Figure 3:**
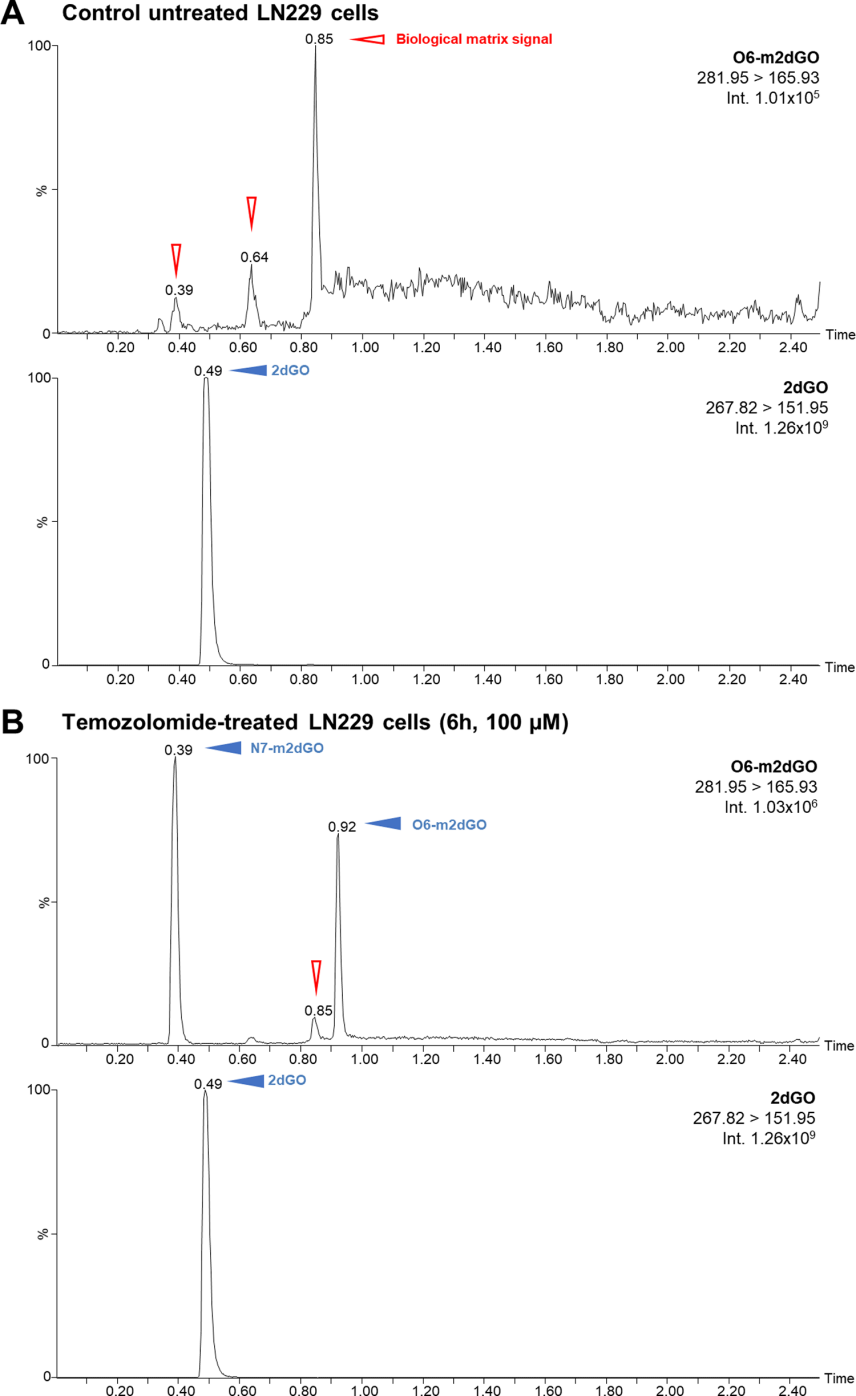
Typical chromatograms of 2dGO and O6-m2dGO observed for digested DNA from untreated GBM cells (LN229 cells) (A) and for digested DNA from LN229 cells treated with 100 μM TMZ for 6 h (B). Filled blue arrows are pointed toward analyte signals and empty red arrows toward biological matrix signals.

Analytical development steps (Figure 2.1–3) consisted of: (1) characterizing the selected compounds and their fragments by MS and MS/MS (Supporting Information, Figure S2), (2) optimizing instrument parameters for their analysis (Supporting Information, Table S1), and (3) selecting and optimizing the most adapted chromatographic approach and MRM transitions to separate N7-m2dGO from O6-m2dGO and specifically quantify O6-m2dGO (Supporting Information, section “Supplementary Results,” and Table 2). The detailed results of initial developments are described in Supporting Information, section “Supplementary Results.” Although important in-source/early post-source decay events were observed during compound characterization (Supporting Information, Figure S2), the C18-LC-MRM/MS assay was optimized with transitions containing the parent species as shown in Table 2. This strategy allows for a high flexibility in further sample preparation and the development of workflows without RNA removal.

### Preparation of Cell Samples, Calibration Standards, and Quality Controls (Figure 2.4)

#### Matrix Effect Determination and Choice of the Matrix for Calibration Standards and Quality Controls

It is usually recommended by the regulatory agencies to prepare CAL and QC samples in the same matrix as the analyzed samples (i.e., here, extracted and digested DNA from a GBM cell line). However, in the present case, the preparation of CALs for O6-m2dGO in biological matrix would necessitate creating large amounts of digested DNA, which would represent very high costs and time for daily assays, including cost and working time for cell cultures, DNA extraction, and DNA digestion. Additionally, the designed assay involved the evaluation of responses of 2dGO in order to normalize the responses of O6-m2dGO to the unmethylated counterpart in DNA. In that context, the use of digested DNA was thus not possible for the creation of calibration curves of 2dGO because it is an endogenous compound. For the quantification of endogenous compounds, the regulation allows the use of alternative matrices (i.e., surrogate matrix approach) as long as the matrix effect between the biological matrix and the alternative matrix is reproducible.^11^ Therefore, two alternative matrices were compared to digested DNA from GBM cells: (i) the digestion buffer from the DNA digestion kit and (ii) the eluent (H_2_O/ACN 95:5 (v/v) 0.1% FA) used for the final samples to be analyzed, as previously described.^14^ The objective was to test whether the calibration curves could be performed in eluent to quantify cell samples. We verified whether responses obtained were the same with CALs prepared in eluent, DNA digestion buffer, and digested DNA. Calibration curves were prepared by spiking increasing concentrations of O6-m2dGO (from 50 pg/mL to 1 ng/mL over five non-zero levels) while keeping its IS at fixed concentration in digested DNA, digestion buffer, and eluent. All levels were prepared in triplicates for precision calculations (% CV), and matrix factors were calculated between digested DNA and digestion buffer, and between digested DNA and eluent for both O6-m2dGO (MF_O6_) and d3-O6-m2dGO (MF_IS_). The normalized matrix factors (MF_norm_ = MF_O6_/MF_IS_) and related precisions were then calculated (Table 3). Calculated MF_norm_ revealed that LC–MS signals of both O6-m2dGO and d3-O6-m2dGO were similarly affected by the changes of matrix, and the normalization by d3-O6-m2dGO can thus correct the matrix effect. Both alternative matrices gave comparable results to digested DNA (MF_norm_ ∼ 1) and could then be used for the preparation of CALs and QCs. For subsequent developments, the CALs and QCs were then prepared in eluent to avoid the additional costs related to the use of additional DNA digestion buffer.

**Table 3:** Summary of Matrix Factor (MF) Calculations for O6-m2dGO, Its Deuterated Internal Standard (d3-O6-m2dGO), and the Normalization^a^

	O6-m2dGO		d3-O6-m2dGO		normalized	
	mean MF_O6_	precision (% CV)	mean MF_IS_	precision (% CV)	mean MF_norm_	precision (% CV)
digestion buffer vs digested DNA	0.985	6	0.977	7	1.000	3
eluent vs digested DNA	1.968	5	1.971	4	0.996	3

a Mean values were calculated with the obtained matrix factors for each triplicate of the five non-zero calibration levels and related precision calculations.

MF_O6_ without normalization showed that signal of O6-m2dGO was 50% lower in digested DNA than in eluent (Table 3, O6-m2dGO). If eluent can be used as an alternative matrix, thanks to the normalization by the IS, for method validation and further quantification, blank digested DNA will need to be used to determine the minimum area required for the lower limit of quantification (LLOQ).

#### Creation of the Cell Model

For the development and validation of the method, a cell model had to be established to allow comparative analyses between untreated cells and cells treated with TMZ at different concentrations, as well as the detection and reproducible quantification of O6-m2dGO. The LN229 cells, the selected biological model for the detection of alkylated guanines, needed to be exposed to TMZ for harmless periods, avoiding apoptosis (DNA degradation) or senescence. On the other hand, drug concentrations had to be high enough to ensure detectable adduct levels. The preliminary biological tests had shown that three days of constant TMZ exposure were required to affect proliferation (Supporting Information, section “Supplementary Results” and Figure S3A). Accordingly, a 6-h exposure to 100 μM TMZ was chosen for the preparation of drug-treated samples. The treated cells were subsequently analyzed using the optimized LC-MRM-MS method and results displayed clear signals for 2dGO and O6-m2dGO, thus indicating an effective action of TMZ even within 6 h (Figure 3).

An intense signal for N7-m2dGO was also observed on the chromatogram corresponding to the O6-m2dGO MRM transition from TMZ-treated cells. N7-m2dGO was clearly separated from O6-m2dGO signal, thus confirming results obtained during early method developments (Supporting Information, section “Supplementary Results”).

This model was thus applied for the preparation of untreated and TMZ-treated cells for method validation.

### Bioanalytical Method Validation

Validation according to BMV regulatory guidelines is necessary for bioanalytical assays developed to quantify drugs or molecular surrogates of their action.^11^ Although this is only a requirement for the analysis of clinical samples, BMV for preclinical samples also ensures the reliability and reproducibility of data produced in preclinical research and its transferability. However, the existing guidelines are not always fully applicable and need to be adapted to the specific requirements of the assays, e.g., for the analysis of endogenous compounds or of modifications of endogenous compounds. In this study, the main focus was to quantify O6-m2dGO in TMZ-treated cell samples and evaluate the possibility to quantify 2dGO. The present BMV was thus focused on the assay for O6-m2dGO, and QCs were only prepared for O6-m2dGO. The BMV was designed to assess the reliability of several critical aspects (Figure 1, Table 4).

(i) The method linearity was assessed using duplicate CALs at seven non-zero concentration levels and one zero level (i.e., CAL with only the IS). CALs were prepared in eluent (H_2_O/ACN 95:5 (v/v) 0.1% FA) (Figure 1A). The accuracy and precision of the analytical measurement was evaluated using QC samples at four concentration levels, each with six biological replicates. Two QC series were prepared: one in eluent (Figure 1B) and one in digested DNA from control LN229 cell samples (Figure 1C).
(ii) Reproducibility of the matrix effect between the selected alternative matrix (i.e., eluent) and the biological matrix (i.e., digested DNA from LN229 cells) was assessed by comparing the QC samples prepared in eluent and in digested DNA for the accuracy and precision evaluation (Figure 1B,C).
(iii) The specificity was tested using digested DNA from control cell samples in six biological replicates (i.e., from six different cell cultures). These control samples were also used to determine the minimum O6-m2dGO signal required at the LLOQ (Figure 1D).
(iv) The reproducibility of TMZ incorporation in LN229 cell lines after 6 h of incubation at three different concentrations was evaluated using six biological replicates for each TMZ concentration level (Figure 1D).
(v) Digestion recovery was evaluated by comparing the 2dGO quantification in samples to expected concentration from the initial amounts of DNA (i.e., 5 μg DNA/sample) (Figure 1C,D).

**Table 4:** Parameters to be Evaluated during Prevalidation and Validation Batches for the Quantification of O6-m2dGO in Digested DNA from GBM Cells Treated with TMZ^a^

	prevalidation batch	validation batch
lower limit of quantification reevaluation	X	
check of linearity range O6-m2dGO	X	
check of linearity range 2dGO	X	X
evaluation of 2dGO dilution strategies		X
method specificity evaluation (6 blank biological matrices)	X	X
precision and accuracy determination (6 biological replicates for each QC level)	X	X
O6-m2dGO matrix factor determination (1 QC series in eluent and 1 in digested DNA)	X	X
reproducibility of TMZ incorporation in DNA (6 biological replicates for each TMZ concentration level)	X	X
O6-mdGO/2dGO ratio calculations		X
digestion recovery determination		X

a 2dGO: 2′-deoxyguanosine; O6-m2dGO: O6-methyl-2dGO; QC: quality control sample; TMZ: temozolomide.

It should be noted that in classical drug quantification assays the recovery and recovery reproducibility have to be evaluated over the concentration range in biological replicates. Since no eligible classical QC samples exist for this type of assay (analytes being a modified form of an endogenous compound embedded in the endogenous polymer DNA), recovery of the process could not be evaluated using classical methods. However, since all digested DNA samples were processed from the same amount of DNA (i.e., 5 μg) and the reproducibility of TMZ treatment and O6-m2dGO quantification were both tested in multiple biological replicates, obtaining satisfactory precision data would demonstrate the reproducibility of the process.

**Figure 4:**
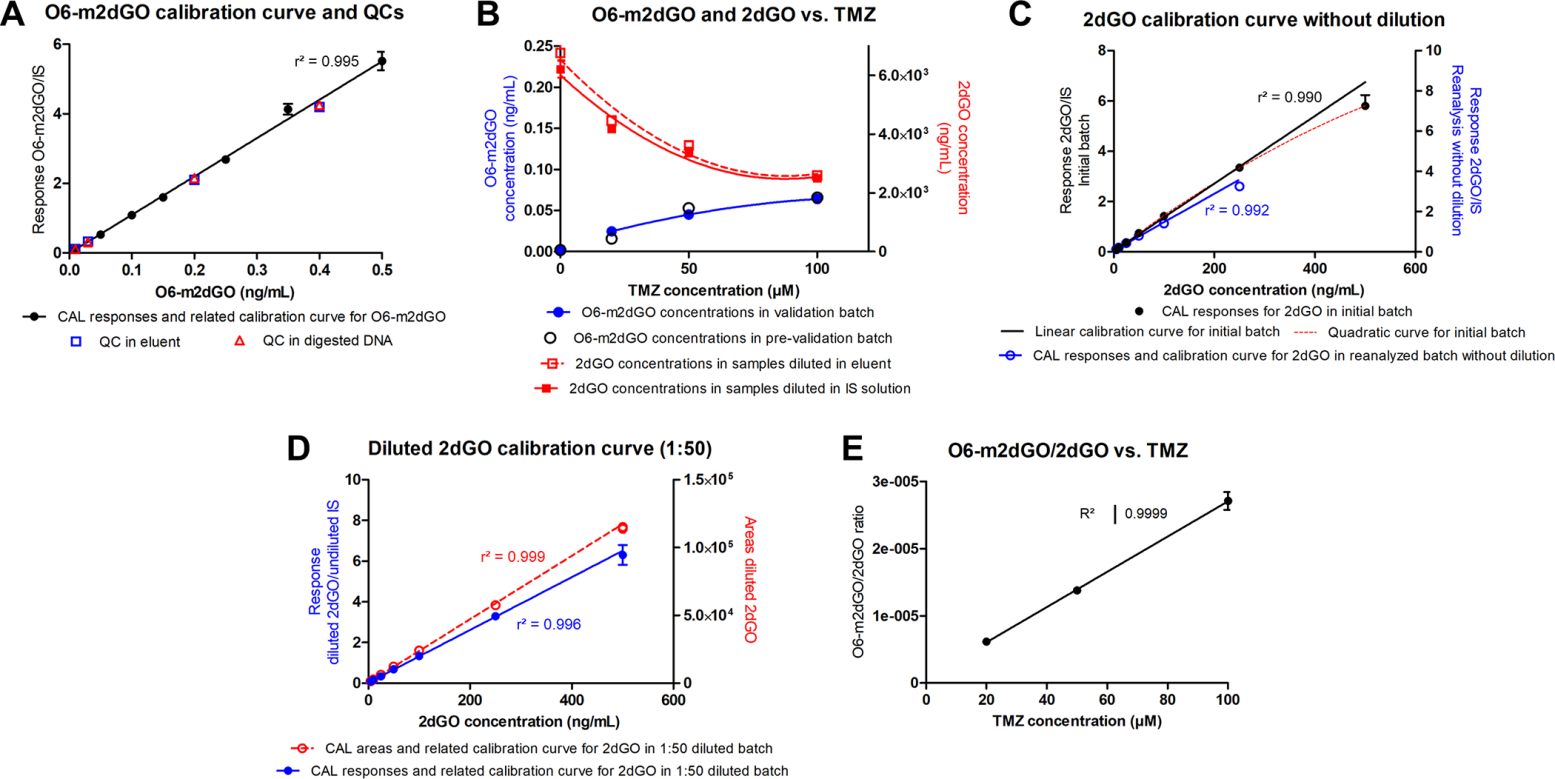
Calibration curve in eluent obtained for the quantification of O6-m2dGO in the validation batch with quality controls (QCs) prepared in eluent and in digested DNA (A). Correlation curves between TMZ concentration applied to cell cultures and related 2dGO and O6-m2dGO concentrations found in digested DNA from control and treated cell samples in the prevalidation batch (empty black circles) and in the validation batch (blue circles for O6-m2dGO and red squares for 2dGO) (B). Calibration curves in eluent obtained for the quantification of 2dGO in the validation batch without dilution with the linear calibration curve (plain black line) and the quadratic calibration curve (red dotted line) highlighting the 2dGO response saturation from 250 ng/mL in initial batch, and linear calibration curve in reanalyzed batch (blue circles and line) with 250 ng/mL as the new upper limit of quantification (C). Calibration curves in eluent obtained for the quantification of 2dGO after 1:50 dilution of CALs using 2dGO responses (blue circles and line) and 2dGO areas (red circles and dotted line) (D). Relationship of O6-m2dGO/2dGO ratios with applied TMZ concentration in LN229 cells (E). All response and concentration values are mean values displayed with standard deviations bars, which can be sometimes too low to appear on the graphs.

These parameters were first fully assessed in a prevalidation batch aiming to choose the final concentration range of O6-m2dGO to be validated. Prevalidation results are further detailed in Figure 4 and in Supporting Information, section “Supplementary Results.”

#### Validation Batch

The validation batch was performed using the concentrations of CAL and QC samples listed in Table 1. After the prevalidation batch, CAL A (i.e., LLOQ) was adjusted to 0.010 ng/mL and new QC samples were created (LLOQ = 0.010 ng/mL and low-level QC (LQC) = 0.030 ng/mL). New cell samples were created following the same process as for the prevalidation batch to get six biological replicates of each TMZ-treated cell model, and biological replicates of untreated cells.

All aspects of the validation batch for O6-m2dGO quantification were validated with a linearity of *r*^2^ = 0.995 over the calibration range 0.010–0.500 ng/mL (Figure 4A). All (100%) calibration points and 93.7% of all QCs including LLOQ (95.8% of QCs in eluent and 91.7% of QCs in digested DNA) met the required accuracy and precision criteria (i.e., within ±15% bias or ±20% bias at LLOQ, and <15% CV or <20% CV at LLOQ, respectively), and all concentration levels were accepted (i.e., all or all but one replicate per level were accepted) (Table 5). The analysis of digested DNA from control cell samples showed that the minimum area for O6-m2dGO at LLOQ should be 55 arbitrary units (a.u.), and the minimum normalized response should be 0.027. All treated cell samples, CALs, QCs in eluent, and QCs in digested DNA were above this threshold. Matrix effect calculations between high-level QC (HQC), middle-level QC (MQC), LQC, and LLOQ in eluent and in digested DNA in the validation (Table 5) confirmed the results obtained during method development with the reproducible normalized matrix factor over the four concentration levels (mean MF_norm_ = 0.981 with 5.1% CV).

**Table 5:** Summary of Validation Results for Accuracy, Precision, and Matrix Effect of Calibration Standards and Quality Control Samples^a^

	in eluent			in digested DNA			matrix effect	
	accepted	accuracy (% bias)	precision (% CV)	accepted	accuracy (% bias)	precision (% CV)	MF_norm_	precision (% CV)
CALs	14/14	–10.1 to 11.3	0–10					
LLOQ	5/6	4.1 to 16.0	6.4	5/6	–10.1 to 19.8	12.7	0.969	16.7
LQC	5/6	–0.9 to 10.9	8.4	6/6	–9.9 to 6.2	6.7	0.915	12.0
MQC	6/6	–8.3 to −1.0	3.1	6/6	–7.8 to 4.5	3.1	1.026	5.0
HQC	6/6	–8.0 to 0.2	3.3	6/6	–6.3 to 1.3	3.0	1.014	3.1

a CV: coefficient of variation; CAL: calibration standard; LLOQ: lower limit of quantification; QC: quality control; LQC: low-level QC (3 × LLOQ); MQC: middle-level QC (30–50% calibration range); HQC: high-level QC (at least 75% ULOQ). Accuracy (% bias) and precision (% CV) ranges obtained for each concentration level are displayed by giving the lowest and the highest obtained values.

Additionally, concentrations obtained for the six biological replicates of TMZ-treated cells displayed good reproducibility of TMZ incorporation in DNA with an intrabatch precision of 14% CV for each of the three concentrations level. Evolution of O6-m2dG concentration with TMZ concentration showed a good correlation with a visible saturation of TMZ effect at 100 μM (Figure 4B). LN229 cells exposed to 20, 50, and 100 μM TMZ for the validation batch displayed similar concentrations as in the prevalidation batch (Figure 4B), more particularly for cells treated with 50 and 100 μM (interbatch precisions of 25, 4.2, and 0.2% CV for cells treated with 20, 50, and 100 μM TMZ, respectively). Altogether, these results obtained from different biological replicates, on different weeks, and with different operators revealed the robustness of the developed assay to monitor the concentration of O6-m2dGO in TMZ-exposed cell lines.

To calculate the ratio O6- m2dGO/2dGO in DNA from cells exposed to TMZ, known concentrations of 2dGO were also measured in the CALs. As in the prevalidation batch, a saturation of the signal of 2dGO was observed in the calibration curve from 250 ng/mL (Figure 4C, black curve). As designed using the results from the prevalidation batch (Supporting Information, section “Supplementary Results”), two strategies were applied for the reanalysis of the validation batch to quantify 2dGO: (i) 1:50 dilution only of QC and cell samples while maintaining the same IS concentration as in the CALs (Figure 4C), and (ii) 1:50 dilution of CALs, QCs, and cell samples (Figure 4D). With the first strategy, concentrations of 2dGO in QCs prepared in digested DNA could be calculated using the computed calibration curve on the range 5–250 ng/mL from reanalyzed undiluted CALs (Figure 4C, blue curve), together with 2dGO concentrations in control and TMZ-treated LN229 cell samples. Responses from QCs and cell samples ranged from 0.6 to 2.0, thus falling within the linearity range of the method (Figure 4C, blue curve) and allowing the accurate determination of diluted 2dGO concentrations. With the second strategy, only concentrations of 2dGO in control and TMZ-treated LN229 cell samples could be calculated using the computed calibration curve (Figure 4D, blue curve). With this strategy, no IS was added to QCs prepared in digested DNA, it was thus not possible to use responses to calculate 2dGO concentrations in QCs. A second calibration curve was then computed with Prism software (GraphPad Software, Boston, MA, USA) for this strategy (Figure 4D, red dotted curve) using only 2dGO LC peak areas, and 2dGO concentrations in QCs were estimated using this area-based calibration curve. Overall, because all cell samples, QCs, and CALs were diluted, 2dGO responses and areas remained outside the calibration range (up to 13-fold higher in cell samples compared to CALs). However, the method was proven linear over the full calibration range from 5 to 500 ng/mL for both response-based and area-based calibration curves (Figure 4D), and both calibration models were used to estimate 2dGO concentrations in QCs and cell samples and compare data with those from the first dilution strategy. Overall, the two dilution strategies resulted in similar estimations of 2dGO in QCs prepared in digested DNA, and in untreated and TMZ-treated cell samples, irrespective of whether the response-based or the area-based calibration curves were used for calculations (Table 6, Figure 4B, red squares). 2dGO concentrations decreased with the increase of O6-m2dGO and TMZ concentrations (Figure 4B), and related mean O6-m2dGO/2dGO ratios displayed a linear correlation with the applied TMZ concentration (Figure 4E).

**Table 6:** Summary of Quantification Results for O6-m2dGO and 2dGO in TMZ-Treated LN229 Cell Lines^a^

TMZ (μM)	O6-m2dGO^b^ (ng/mL)		2dGO^c^ (ng/mL)		O6-m2dGO/2dGO	
	mean	% CV	mean	% CV	mean	% CV
20	0.027	13.7	4321.3	7.4 (8.6/6.5/7.7)^d^	6.15 × 10^6^	8.0
50	0.049	14.3	3521.8	10.4 (10.5/10.9/10.7)	1.38 × 10^5^	6.0
100	0.069	13.6	2558.6	6.6 (6.4/8.7/6.5)	2.71 × 10^5^	11.9

a 2dGO: 2′-deoxyguanosine; % CV: coefficient of variation/precision; O6-m2dGO: O6-methyl-2dGO; TMZ: temozolomide.

b Mean values for O6-m2dGO were calculated from the six biological replicates analyzed in the undiluted validation batch.

c Mean values for 2dGO were calculated from the six biological replicates for each one of the three applied strategies: (i) response-based quantification from the 1:50 dilution of quality controls and cell samples with IS solution, (ii) response-based quantification from the 1:50 dilution of CALs, QCs, and cell samples with eluent only, and (iii) area-based quantification from the 1:50 dilution of CALs, QCs, and cell samples with eluent only.

d Coefficients of variation for 2dGO calculated concentrations are given as follows: “mean % CV (% CV of the response-based strategy from 1/50 dilution with the IS / % CV of the response-based strategy from 1/50 dilution in eluent only/ % CV of the area-based strategy from 1/50 dilution in eluent only)”.

These results indicated that both strategies were valid for 2dGO quantification. For expected 2dGO concentrations outside the calibration range, the first strategy should be used (i.e., dilution of samples while maintaining the same IS concentration as in the initial CALs) to ensure reliable quantification. Alternatively, additional points of calibration could be added including an upper limit of quantification (ULOQ) above the measured concentration of 2dGO (e.g., 1000, 5000, and 10,000 ng/mL). In our study, the ULOQ used to ensure method linearity was 250 ng/mL, which would correspond to a maximal theoretical 2dGO concentration before dilution of 12,500 ng/mL. The alternative additional calibration points up to 10,000 ng/mL before dilution would thus fit in the linearity range.

Additionally, digestion recovery could also be estimated using 2dGO quantification results from untreated cells. Human DNA comprises about 20% guanine, 20% cytosine, 30% adenine, and 30% thymine. From here, it could be calculated that 5 μg of DNA would comprise 1080 ng of 2dGO and thus, it could be estimated that the DNA digestion process had a recovery of 21%.

## Discussion

In vitro models are ideal for initial pharmacological tests of new drug candidates in GBM. Because TMZ remains the standard of care for the treatment of GBM and new drugs under development are often used in combination with TMZ, monitoring the direct pharmacodynamic effects of TMZ in cells would be very valuable for early drug development.

The developed bioanalytical assay is intended for research applications to monitor the molecular pharmacodynamic effects of TMZ in vitro when the drug is tested on a cell line alone or in combination with other drug candidates against GBM. Currently, O6-m2dGO is considered to be the biologically active modification of 2dGO induced by TMZ. We aimed to quantify these two compounds while ensuring the separation of O6- from N7-m2dGO species.

Validation of assays is always recommended in drug development and allows the detection of artifacts or technical variations that would hamper the achievement of reliable, interpretable quantification results. The International Council for Harmonisation (ICH) recently edited BMV guidelines for the quantification of drugs in biological matrices, combining recommendations from the US Food and Drug Administration (FDA) and the European Medicines Agency (EMA).^11,15,16^ In the present context, the main objective of the validation was to demonstrate that the alkylation effect of TMZ on 2dGO, together with the overall sample preparation procedure, was reproducible between different samples and using different TMZ concentrations. Thus, the developed assay aimed to monitor chemical modifications induced by a drug metabolite in an endogenous structure, i.e., DNA. Therefore, the regulatory guidelines for drug quantification were not directly applicable for all commonly evaluated parameters (e.g., assessment of analyte recovery), and the validation procedure had to be adapted to account for the specific framework of the method. The DNA digestion yield could be estimated to 21% during this validation procedure based on known quantities of DNA before digestion and corresponding 2dGO concentrations in processed samples. This information might be important when the absolute concentrations of O6-m2dGO are to be determined, but less important when O6-m2dGO/2dGO ratios are of primary interest. Even more important for further applications of the assay was to ensure that the entire workflow (i.e., TMZ absorption, DNA methylation, DNA extraction, and DNA digestion) resulted in reproducible methylation of 2dGO, even when different concentration of TMZ were used. The adapted validation procedure showed, through the treatment of biological replicates, that good precision values were obtained for all concentration levels (i.e., ≤ 25% CV), demonstrating the robustness of the present assay. In addition, the calculated O6-m2dGO/2dGO ratios were shown to have a linear relationship with the applied TMZ concentration.

Future applications may necessitate to adapt exposure time and TMZ concentration to specific needs of the individual research projects. For example, the procedure would need to be adapted to allow quantification of O6-m2dGO and 2dGO in lower cell numbers or after exposure to lower TMZ concentrations. In that case, similar validation of downscaling batches would be performed prior to application to cell samples. The method also provides guidance for future development of quantification methods in tissues. The present quantification method was developed for DNA quantities (i.e., 5 μg DNA) from an equivalent of approximately 2–4 million LN229 cells. In tissue sections of diffuse GBM, only a very limited number of cells can be found from which DNA extraction may not be possible. Direct digestion methods can be considered for the quantification of O6-m2dGO, 2dGO, as well as other nucleosides such as thymine or cytosine, for the mass spectrometric estimation of DNA quantities.

## Conclusions

The aim of the present study was to develop and partially validate a method to monitor the biologically active chemical effect of TMZ on exposed GBM cell lines. A bioanalytical assay was developed for the quantification of O6-m2dGO. This is particularly useful for preclinical in vitro testing of new GBM drugs to be further investigated in combination with TMZ in clinical trials. These developments are also informative first steps for further implementation of downscaled assays using lower cell numbers and/or lower TMZ concentrations, either in vitro with cell culture assays or in vivo using animal models. Besides the overall interest for personalized medicine applications, the development and validation of methods for the quantification of DNA adducts would find a high interest in the field of chemical toxicology.^17^

## Supporting Information Available

Description of standard solution preparation and evaluation of biological effects of TMZ on LN229 (Supplementary Materials and Methods). Description of LC-MRM/MS method development and optimization, effects of TMZ on cell abundance and proliferation, and BMV (Supplementary Results) (PDF)

## Abbreviations

2dGO: 2′-deoxyguanosine
2dNetGO: 2′-deoxy-N-ethyl-guanosine
BMV: bioanalytical method validation
BrdU: bromodeoxyuridine
CAL: calibration standards
CID: collision induced dissociation
d3-O6-m2dGO: d3-O6-methyl-2′-deoxyguanosine
DI-MS: desorption/ionization mass spectrometry
DMEM: Dulbecco’s Modified Eagle’s Medium
EMA: European Medicines Agency
ESI: electrospray ionization
FCS: fetal calf serum
FDA: Food and Drug Administration
FA: formic acid
GBM: glioblastoma
HILIC: hydrophilic-interaction liquid chromatography
HQC: high-level quality control
HR-MS: high-resolution mass spectrometry
ICH: International Council for Harmonisation
IS: internal standard
LC–MS/MS: liquid chromatography–tandem mass spectrometry
LQC: low-level quality control
LLOQ: lower limit of quantification
MS: mass spectrometry
MGMT: methylguanine methyl transferase
MQC: middle-level quality control
MMR: mismatch repair
MRM: multiple reaction monitoring
N2-m2dGO: N2-methyl-2′-deoxyguanosine
N7-mG: N7-methylguanine
O6-mG: O6-methylguanine
O6-m2dGO: O6-methyl-2′-deoxyguanosine
PSD: post-source decay
QC: quality control
SEM: standard error of the mean
TMZ: temozolomide
UPLC: ultra-performance liquid chromatography
ULOQ: upper limit of quantification.
