# Supplementary material for: Quantification of Biologically Active DNA Alkylation in Temozolomide-Exposed Glioblastoma Cell Lines by Ultra-Performance Liquid Chromatography–Tandem Mass Spectrometry: Method Development and Recommendations for Validation
Source: ACS Omega. 2023 Jun 23;8(26):23695–705. doi: 10.1021/acsomega.3c01818 (PMC10324051; doi:10.1021/acsomega.3c01818)
Supplement: Supplementary file 1 — ao3c01818_si_001.pdf [file ao3c01818_si_001.pdf]

Supplementary information

Quantification of biologically-active DNA alkylation in temozolomide-exposed glioblastoma cell lines by ultra-performance liquid chromatography-tandem mass spectrometry: method development and recommendations for validation.

Margaux Fresnais[1], Ina Jung[1], Uli B. Klein[1], Dirk Theile[1], Siwen Liang[1], Walter E. Haefeli[1], Jürgen Burhenne[1], Rémi Longuespée[1,*]

[1] Department of Clinical Pharmacology and Pharmacoepidemiology, Heidelberg University Hospital, Im Neuenheimer Feld 410, 69120 Heidelberg, Germany.

*Corresponding author: remi.longuespee@med.uni-heidelberg.de

Supplementary materials and methods

Standard solutions preparation

Stock solutions of 2'-deoxyguanosine (2dGO), O6-methyl-2'-deoxyguanosine (O6-m2dGO), 2'-deoxy-N-ethyl-guanosine (2dNetGO), d3-labeled O6-m2dGO (d3-O6-m2dGO), N7-methylguanine (N7-mG), O6-methylguanine (O6-mG), and N2-methyl-2'-deoxyguanosine (N2-m2dGO) were prepared in MeOH/H_2O 50:50 (v/v) at 1.56 mg/mL, 302 µg/mL, 510 µg/mL, 250 µg/mL, 1.5 mg/mL, 516 µg/mL, and 540 µg/mL, respectively.

For mass spectrometric specificity characterization in high-resolution mass spectrometry (HR-MS) (see **Materials and methods section** in the main document), sub-stock solutions at 10 µg/mL were prepared for direct infusion. For tuning of multiple reaction monitoring (MRM) parameters and liquid chromatography (LC) optimization, sub-stock solutions at 1 µg/mL were prepared for 2dGO, O6-m2dGO, d3-O6-m2dGO, and N7-mG, and the sub-stock solution at 10 µg/mL were used for 2dNetGO and N2-m2dGO. Solutions were prepared by mixing the targeted compounds either in ACN/H_2O 95:5 (v/v) 0.1 % formic acid (FA) for methods using a hydrophilic-interaction liquid chromatography (HILIC) column or in H_2O/ACN 95:5 (v/v) 0.1 % FA (LC eluent) for methods using C18 LC column.

Evaluation of biological effects of temozolomide on LN229

A total of 10 000 cells/well were seeded in 96-well plates and allowed to adhere overnight. The next day, the cell culture medium was replaced with fresh medium containing different concentrations of temozolomide (TMZ). After three to six days of constant drug exposure, cell abundance was measured by staining attached cells with crystal violet. Drug effects were normalized to untreated control wells of the same treatment period. Because the relative cell abundance is the net effect of direct cytotoxicity and proliferation inhibition, the latter was

additionally evaluated by recording the incorporation of bromodeoxyuridine (BrdU) into replicated DNA during TMZ exposure. Based on the manufacturer's instructions and our previous protocol [1], LN299 cells (1000 cells/well) were seeded into 96-well plates and allowed to adhere overnight. The next morning, medium was replaced by TMZ-containing medium, and 20 µL of BrdU solution was added to the wells. BrdU incorporation after three, four, or five days of TMZ exposure was determined by washing, fixing, and exposing the wells to a mouse anti-BrdU antibody (1 h at room temperature), followed by exposure to a peroxidase goat anti-mouse antibody (30 min at room temperature). The peroxidase oxidizes the transparent tetramethylbenzidine (added to the wells for 15 min) to a bluish compound. This reaction is stopped by adding the stop solution from the kit, which turns the bluish color into yellow. Its absorption was recorded at 450 nm. BrdU incorporation was normalized to values recorded at t = 0. Because three days of constant TMZ exposure hardly affected cell abundance or proliferation (**Figure S3**), all subsequent analytical developments were performed after a shorter exposure, set at 6 h to allow the development of a rapid method.

Supplementary results

LC-MRM/MS method development and optimization

Choice of standards and internal standards

The two main standards used in this study were 2dGO (**Supplementary Figure S1.A**) and O6-m2dGO (**Supplementary Figure S1.B**). The classical fragmentation pattern of nucleosides takes place between the ribose and the purine moieties and the guanine is necessary for the quantification of the transition. For internal standards (IS), we aimed at finding compounds labelled at the guanine moiety. Therefore, IS used were 2dNetGO (**Supplementary Figure S1.D**) and d3-O6-m2dGO (**Supplementary Figure S1.E**). N7-methyl-2'-deoxyguanosine (N7-

m2dGO) (**Supplementary Figure S1.C**) was not commercially available; therefore, different surrogates were used for fragments characterization and chromatographic separation. N7-mG (**Supplementary Figure S1.H**) was used to extrapolate possible fragments of N7-m2dGO. Couples N2-m2dGO (**Supplementary Figure S1.F**)/ O6-m2dGO (**Supplementary Figure S1.B**) and N7-mG (**Supplementary Figure S1.H**)/O6-mG (**Supplementary Figure S1.G**) were used to extrapolate chromatographic separation of O6-m2dGO (**Supplementary Figure S1.B**) and N7-m2dGO (**Supplementary Figure S1.C**). O6-m2dGO and N7-m2dGO have xlogP values of 0.1 and -1.6, respectively. Since N2-m2dGO has an xlogP value of -0.5, the hydrophobicity difference between O6- and N7-m2dGO is higher (∆xlogP = 1.7) than between O6- and N2-m2dGO (∆xlogP = 0.6). Therefore, the ability to separate these two compounds would guarantee the ability to separate O6- from N7-m2dGO. The separation of O6- and N7-mG with respective xlogP values of -0.3 and -1.1 (∆xlogP = 0.8) would also ensure the separation of O6- and N7-m2dGO.

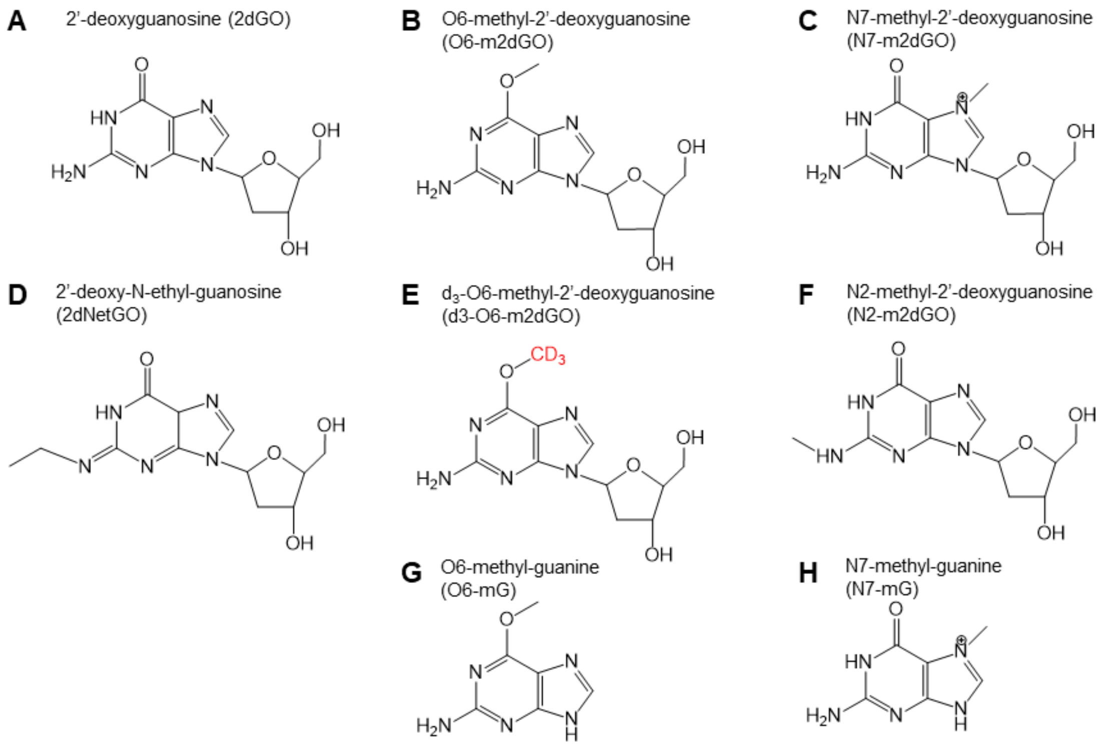

Supplementary Figure S1 – Chemical structures of the unmodified nucleoside 2'-deoxyguanosine (2dGO, A), its biologically active methylated form O6-methyl-2'-deoxyguanosine (O6-m2dGO, B), and its inactive main methylated form N7-methyl-2'-deoxyguanosine (N7-m2dGO, C), together with the selected internal standards for 2dGO, 2'-deoxy-N-ethyl-guanosine (2dNetGO, D), and for O6-m2dGO, d_3-O6-methyl-2'-deoxyguanosine (d3-O6-m2dGO, E). As N7-m2dGO was not commercially available, additional compounds such as N2-methyl-2'-deoxyguanosine (N2-m2dGO, F), O6-methyl-guanine (O6-mG, G), and N7-methyl-guanine (N7-mG, H) were studied for further investigation of O6-species and N7-species chromatographic separation and for fragment characterization of N7-m2dGO.

MS and MS/MS characterization

Previous observations with desorption/ionization (DI)-MS indicated that in-source decay (ISD) or early post-source decay (PSD) events could be observed for 2dGO and its methylated forms [2]. For the characterization of the compounds in electrospray ionization (ESI), the possible occurrence of these events was verified using HR-MS. The results indicated that similar PSD events could be observed in ESI using HR-MS (**Supplementary Figure S2**) and also on a triple-quadrupole mass spectrometer. The optimal MRM parameters (cone voltage and collision energy) were tuned using the Intellistart tool of the MassLynx software (Waters Corp, Milford, MA, USA) and the list of the six most intense MRM transitions was retrieved for each compound (**Supplementary Table 2**).

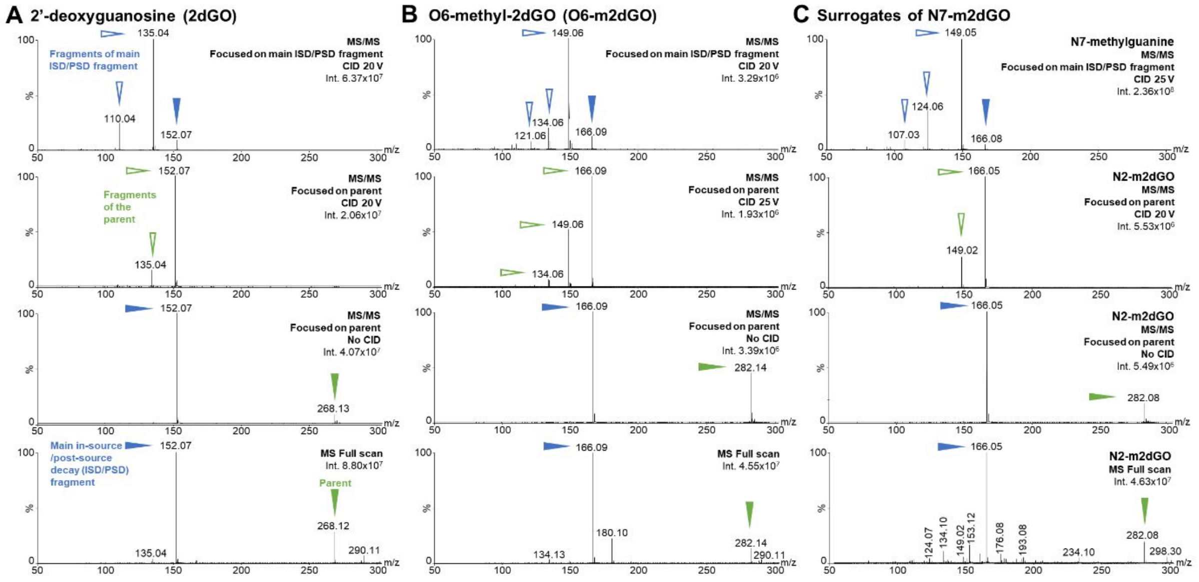

Supplementary Figure S2 – Mass spectrometry (MS) and MS/MS spectra of 2'-deoxyguanosine (2dGO, A), O6-methyl-2'-deoxyguanosine (O6-m2dGO, B), surrogates of N7-methyl-2'-deoxyguanosine (C), N2-methyl-2'-deoxyguanosine (N2-m2dGO) and N7-methylguanine (N7-mG). MS spectra of intact parents (2dGO, O6-m2dGO, and N2-m2dGO) were acquired in full scan mode or with the quadrupole focused on the parent mass without collision induced dissociation (CID). MS/MS spectra of 2dGO, O6-m2dGO, and N7-mG were acquired with the quadrupole focused either on the parent mass or on the main post-source decay fragment with CID at 20-25 V. Maximum intensities are given in upper right corner of each spectrum.

Supplementary Table S1 – Optimized multiple reaction monitoring transitions of each studied compound.

Compound	MRM parent (m/z)	Cone voltage (V)	MRM daughter (m/z)	Collision energy (V)
2dGO	267.82 (intact compound)	2	151.95	10
		2	134.92	32
		2	109.90	32
	151.75 (ISD/PSD fragment)	44	109.85	16
		44	106.92	22
		44	134.83	16
O6-m2dGO	281.95 (intact compound)	22	148.90	32
		22	165.93	14
		22	134.14	36
	165.88 (ISD/PSD fragment)	76	148.94	16
		76	134.11	18
		76	109.78	18
2dNetGO	296.01 (intact compound)	8	180.07	12
		8	163.04	32
		8	134.96	40
	179.16 (ISD/PSD fragment)	52	163.01	18
		52	134.92	22
		52	109.95	20
d3-O6-m2dGO	284.95 (intact compound)	18	151.89	32
		18	168.93	14
		18	134.08	36
	168.82 (ISD/PSD fragment)	68	151.90	16
		68	134.08	18
		68	106.82	26
N2-m2dGO*	282.03 (intact compound)	24	166.03	12
		24	149.00	32
		24	109.99	36
	165.90 (ISD/PSD fragment)	52	149.03	22
		52	109.95	24
		52	134.99	18

MRM: multiple reaction monitoring; ISD: in-source decay; PSD: post-source decay; 2dGO: 2’-deoxyguanosine; O6-m2dGO: O6-methyl-2dGO; 2dNetGO: 2’-deoxy-N-ethyl-guanosine; d3-O6-m2dGO: d3-labeled O6-m2dGO; N2-m2dGO: N2-methyl-2dGO.

* Surrogate of N7-methyl-2’-deoxyguanosine (N7-m2dGO).

Supplementary Table S1 shows that a lower cone voltage was required to retrieve the maximum intensities for the transitions using the intact compound as a parent, while higher cone voltages were always required to get maximum intensities for the transitions involving the ISD/PSD fragment as a parent.

Selection of the chromatographic method

Based on the relative polarity of the analytes, two strategies could be investigated for their LC separation: (i) HILIC-based separation, which would seem to be most suitable due to the high polarity of the compounds, and (ii) C18-based separation. The use of HILIC would require starting the LC gradient with nonpolar conditions (highest percentage of ACN in the eluent), while the workflow for DNA extraction and digestion was performed in aqueous solutions and no solvent/buffer exchange was planned to avoid sample and time loss during the process. To achieve optimum LC separation and analyte LC peaks, initial composition of the eluent system and of the washing solvent of the LC injection system should be as close as possible to the sample solvent composition (i.e., high percentage of the water-based eluent). This was confirmed in preliminary tests in which N7- and O6-m2dGO, or N2- and O6-m2dGO were solubilized in H_2O/ACN 95:5 (v/v) 0.1% FA and analyzed by LC-MS/MS with an HILIC column and a C18 column. Under these conditions, it was not possible to separate these compounds with HILIC.

For final optimization of the multiplexed O6-m2dGO/2dGO method, the C18-based LC method detailed in the **Materials and Methods Section** was further applied to the selected transitions of the different compounds to test for the specificity of all possible transitions of O6-m2dGO, N2-m2dGO, and 2dGO.

Assessment of chromatographic and mass spectrometric specificity

For the development of the multiplexed method, the most specific and intense transitions of 2dGO and O6-m2dGO were screened and the separation between 2dGO, N2-m2dGO, and O6-m2dGO was verified. The main compounds of interest (2dGO and O6-m2dGO, and their respective IS, 2dNetGO and d3-O6-m2dGO) were analyzed pure or mixed together using an MS acquisition method that included each of the selected MRM transitions, as tuned by Intellistart (**Table 2**).

The selected transitions corresponded to the ones that could be used for methods omitting RNA removal prior to DNA digestion. Indeed, the selection of parent ions corresponding to unmethylated and methylated 2dGO species would allow them to be distinguished from unmethylated and methylated guanosine species of RNA.

Effects of TMZ on cell abundance and proliferation

The impact of TMZ exposure on cell abundance was evaluated by crystal violet staining. TMZ did not exert direct cytotoxic effects because cell abundance remained unaffected up to 100 µM for three days (**Supplementary Figure S3.A**). The BrdU incorporation assay showed that long exposure times (> 6 d) are required to lower LN299 cell proliferation (**Supplementary Figure S3.B**).

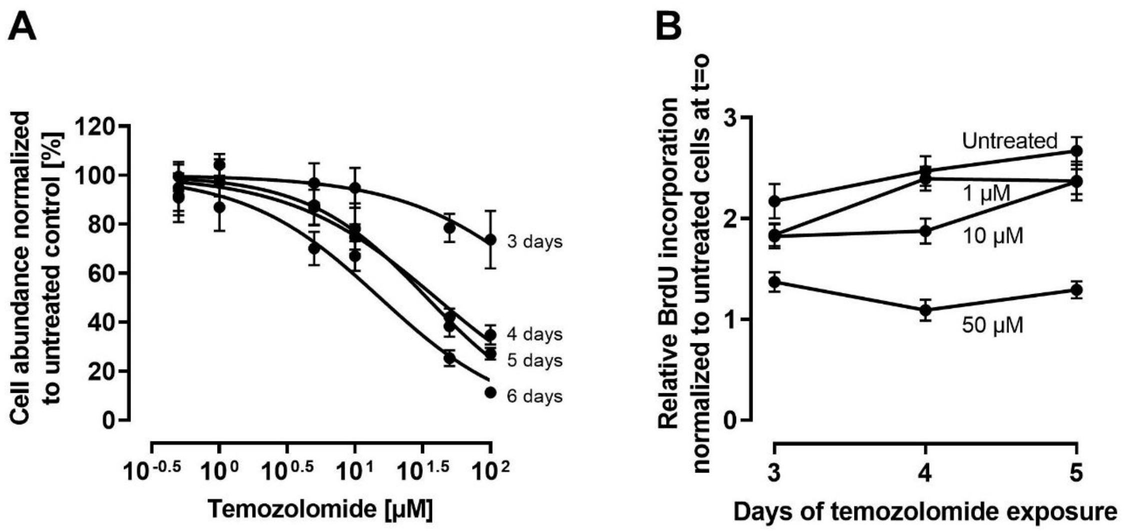

Supplementary Figure S3 – Impact of temozolomide on cell abundance (A) or bromodeoxyuridine (BrdU) incorporation (B) after exposure of LN299 cells to different drug concentrations for variable time periods. Data display mean values with standard error of the mean (SEM) of independent biological triplicates each measured in three to eight technical replicates. Data in part A was fitted according to an Emax model (four parameter-logistic equation; variable slope).

Bioanalytical method validation (BMV)

In this study, the main focus was to quantify O6-m2dGO in TMZ-treated cell samples and evaluate the possibility to quantify 2dGO. The present BMV was thus focused on the assay for O6-m2dGO and quality control samples (QCs) were only prepared for O6-m2dGO. The BMV was designed to assess the reliability of several aspects, as detailed in **Table 3**. These parameters were first fully assessed in a pre-validation batch aiming to choose the final concentration range of O6-m2dGO to be validated.

Pre-validation batch

For the pre-validation batch, duplicate eluent samples of each calibration standard (CAL) level and six replicates of eluent and digested DNA samples of each QC level were prepared

using the O6-m2dGO and 2dGO initial concentrations detailed in **Table 1**, as detailed in **Figure 4** in the main document. The biological replicates of TMZ-treated LN229 cell samples were prepared with 6-h exposure to 0, 20, 50, and 100 µM (**Figure 4.D**). Finally, duplicate samples of eluent and pooled digested DNA were also analyzed as a blank matrix for specificity and evaluation of minimal lower limit of quantification (LLOQ) signal.

The first parameter evaluated in the first batch was specificity. Analyses of digested DNA from control cell samples showed that the minimum area required for the LLOQ samples (i.e., 5-times mean area from blank control samples) was not reached with a LLOQ concentration of 0.005 ng/mL and that the next lowest CAL level (i.e., 0.010 ng/mL) should allow to fulfill this parameter. Additionally, linearity over the concentration range above 0.010 ng/mL was satisfactory with an $r^2 = 0.993$, the final concentration range to be validated was thus set to 0.010-0.500 ng/mL (**Table 1**) with the LLOQ level at 0.010 ng/mL. Together with the CAL levels, the QC levels were also revised for validation, and more particularly LLOQ and low-level QC (LQC) levels as detailed in **Table 1**. In the first batch, different classical parameters of BMV could first be assessed: accuracy and precision (QC samples in eluent and digested DNA), matrix effect (by comparison of QC in eluent and QC in DNA samples), and carry-over (by evaluation of eluent samples analyzed after the CAL H and HQC samples). They globally met the required criteria (precision < 15 % CV, or 20 % CV at LLOQ; and accuracy within ± 15 % bias, or 20 % bias at LLOQ) except for the LLOQ levels and the LQC level in eluent, confirming the need to revise the concentration range of the assay. An additional parameter of inter-sample precision was evaluated in TMZ-treated cells. Unlike assays developed for clinical trials, samples from preclinical developments are generated experimentally, and the precision of quantification results can contribute to the evaluation of the entire process, i.e., from exposure to MS analysis. For cells treated with 20 µM TMZ,

areas were below the threshold calculated from the control cell samples and O6-m2dGO could thus only be estimated in these cell samples. However, responses from cells exposed to 50 and 100 µM TMZ suggested that O6-m2dGO would be quantified in these samples with satisfying inter-sample precision (≤ 20 % CV). The final settings were then validated in one validation batch. In classical drug quantification assays, the recovery and recovery reproducibility have to be evaluated over the concentration range in biological replicates. Since no eligible classical calibration samples exist for this type of assay (analytes being a modified form of an endogenous compound), recovery of the process could not be evaluated using classical methods. However, since all digested DNA samples were processed from the same amount of DNA (i.e., 5 µg) and the reproducibility of the TMZ treatment and O6-m2dGO quantification was tested in multiple biological replicates, the achievement of satisfactory precision data tends to be evidence of the reproducibility of the process.

In the present method development, a secondary goal was to estimate the ratio O6-m2dGO/2dGO in DNA from cells exposed to TMZ. In the calibration samples, known concentrations of 2dGO were also measured. As formerly observed, a saturation was expected over the concentration range of 2dGO, sample dilution was then necessary to quantify 2dGO while avoiding saturation of the MS device. In the first analysis of the pre-validation batch, the saturation of the curve was observed from 250 ng/mL. The pre-validation batch was then diluted 50-fold and analyzed again. The diluted calibration curve over the 2dGO concentration range was linear with an $r^2 = 0.997$. Thus, two strategies were possible for the quantification of 2dG. The first would be to dilute the calibration samples and the samples to quantify 2dGO. When the areas of 2dGO at the upper limit of quantification (ULOQ) are inferior to the area in the samples, it would be at least possible to estimate 2dGO by extrapolation. Alternatively, the second strategy would consist of reanalyzing the

undiluted calibration points and diluting the samples in IS solution to maintain the same IS concentration as in the CAL samples. In this second strategy, the ULOQ would then be the last concentration for which the curve was linear (i.e., 250 ng/mL) and the concentration of 2dGO in samples would be back-calculated using the applied dilution factor. Both strategies were tested in the reanalysis of the validation batch, as detailed in the main document.

References

1. Theile, D.; Ketabi-Kiyanvash, N.; Herold-Mende, C.; Dyckhoff, G.; Efferth, T.; Bertholet, V.; Haefeli, W. E.; Weiss, J., Evaluation of drug transporters' significance for multidrug resistance in head and neck squamous cell carcinoma. *Head Neck* **2011,** *33* (7), 959-68.
2. Fresnais, M.; Jung, I.; Klein, U. B.; Miller, A. K.; Turcan, S.; Haefeli, W. E.; Burhenne, J.; Longuespee, R., Important Requirements for Desorption/Ionization Mass Spectrometric Measurements of Temozolomide-Induced 2'-Deoxyguanosine Methylations in DNA. *Cancers* **2023,** *15* (3).
